# Immune Response of the Pacific Oyster *Magallana gigas* Following Shell Damage

**DOI:** 10.3390/ani16203310

**Published:** 2026-10-21

**Authors:** Daria S. Lavrichenko, Elizaveta A. Bogacheva, Viktoria A. Uppe, Ekaterina A. Vodiasova, Elina S. Chelebieva, Ekaterina S. Kladchenko

**Affiliations:** https://ror.org/03qf4s180 A.O. Kovalevsky Institute of Biology of the Southern Seas of RAS, Moscow 119991, Russia; lavrichenko_daria@ibss-ras.ru (D.S.L.); liza.bogacheva@ibss-ras.ru (E.A.B.); uppeviktoria@ibss-ras.ru (V.A.U.); e.chelebieva@ibss-ras.ru (E.S.C.)

**Keywords:** *Magallana gigas*, shell damage, shell repair, hemocytes, cellular immunity, oyster health

## Abstract

Shell integrity is a key determinant of health in cultured bivalves; however, its damage may affect physiological and immune processes associated with repair. This study evaluated the effects of a single non-penetrating shell damage on cellular immune responses and immune-related gene expression in the mantle of the Pacific oyster *Magallana gigas*. Over 14 days post-damage, reactive oxygen species (ROS) and reactive nitrogen species (RNS) production, phagocytic activity, adhesion, and aggregation of hemocytes were assessed. Relative expression of tumor necrosis factor (TNF-1, TNF-2), interleukin-17 (IL-17-3, IL-17-6), and Toll receptor (Toll-1) genes was also determined in mantle tissue. The damage induced predominantly temporal alterations in hemocyte functional activity. During the first two days, ROS production decreased, while RNS production remained unchanged; phagocytic activity increased on day 1 and decreased on day 2. More prolonged changes were observed in hemocyte adhesion and aggregation. Of the genes examined, only TNF-2 showed a significant change, with decreased expression on day 7. These results show that a single non-penetrating shell damage primarily induces a broad stress-associated physiological response, which transiently modulates cellular immune parameters and local regulation mechanisms. By the end of the experiment, most parameters did not differ from the control, although certain changes persisted during shell repair.

## Simple Summary

Bivalve aquaculture is rapidly expanding to meet the growing demand for animal protein, making the health and resilience of farmed oysters increasingly important. The Pacific oyster *Magallana gigas* is one of the most widely cultivated bivalves, but intensive farming is associated with an increased risk of disease and environmental stress. The shell is the first line of defence against the external environment, but damage to its integrity can increase susceptibility to pathogens and initiate energetically costly repair processes. Because energy resources are limited, investment in shell repair may affect other energy-demanding functions, including immune defence. However, it remains unclear how much of the immune response observed during chronic shell damage is caused directly by the damage itself. Understanding how oysters respond to factors that compromise their health is therefore essential for assessing the resilience of farmed populations.

## 1. Introduction

Driven by the increasing global need for animal protein, bivalve aquaculture is becoming one of the most rapidly developing sectors of the food industry. The Pacific oyster *Magallana gigas* is one of the most widely cultivated bivalve species, owing to its high adaptive potential [1,2]. However, the intensive development of aquaculture is accompanied by an increased risk of diseases in farmed molluscs, which can lead to a decline in their physiological status and mass mortality [3,4]. Oyster health in aquaculture is determined by their ability to resist pathogenic microorganisms and unfavourable environmental stressors [5,6]. In this context, understanding the immune defence mechanisms of *M. gigas* and the factors that may alter the effectiveness of the immune response is essential for assessing the resilience of farmed populations.

Molluscs, like other invertebrates, possess only an innate mechanism of pathogen control and defence, relying on a non-specific immune response [7]. The bivalve’s innate immunity comprises a complex of barrier, cellular, and humoral mechanisms that ensure recognition and elimination of potential pathogens [8,9]. The shell serves as the first line of defence in bivalves, providing not only structural support and predator protection, but also contributing to the maintenance of physiological homeostasis [10]. The next protective barrier is the mantle [11]. Like the shell, the mantle limits direct contact of internal tissues with the environment and participates in mucosal immune defence [12]. The key cellular component of innate immunity in bivalve molluscs is hemocytes—circulating hemolymph cells involved both in recognition and elimination of foreign agents and in response to tissue damage. Hemocytes mediate a wide range of cellular responses, including adhesion, aggregation, phagocytosis, and production of reactive oxygen and nitrogen species [13].

Disruption of shell integrity may lead to changes in barrier properties and increased susceptibility of molluscs to pathogenic microorganisms. Brioukhanov et al. [14] reported that shell damage inflicted by boring sponges is linked to considerable alterations in the oyster gill microbiome. A greater diversity of microfungi was also found on oyster shells infested with the boring sponge compared to healthy shells [15]. In addition to the impairment of barrier function, shell damage triggers repair processes involving mantle cells and hemocytes, which are functionally linked to both immune responses and biomineralization [16,17]. This establishes a close functional link between shell repair and innate immune defence mechanisms. In particular, Xiong et al. [17] demonstrated that during repair of damaged shells in *Pinctada fucata martensii*, changes in the expression of immune-related genes were observed as early as 12 h post-damage, indicating the involvement of immune mechanisms at the early stages of repair.

Although molluscs possess the capacity for shell regeneration [18], the speed and time course of repair differ among species. Zhang et al. [19] reported that in *Ruditapes philippinarum*, the outer shell layer began to form within 5 days after damage, while *Mytilus edulis* required a longer period, attaining a similar degree of shell regeneration only on day 11 of the experiment [20]. Shell repair and the associated biomineralization require significant energy costs [20,21]. Since the energy resources available to the organism are limited and may be further reduced under stress conditions [22], the concurrent support of multiple energetically. Similar functional trade-offs have been demonstrated for various physiological processes in bivalves. For example, Brokordt et al. [23] identified a bioenergetic trade-off between reproduction and immunity, which was accompanied by reduced activity of several immune responses during the reproductive phase. Potential competition for energy resources has also been suggested between immune defence and biomineralization [24]. Therefore, reallocation of resources towards repair of the damaged shell may be accompanied by changes in the activity of other energetically costly functions, including immune defence.

We have previously reported that shell infestation of *M. gigas* by the boring sponge *Pione vastifica* is associated with alterations in several cellular immune parameters. Infected oysters exhibited elevated ROS production and hemocyte phagocytic activity, along with modifications in hemolymph cellular composition [25,26]. Furthermore, tissue-specific changes in biomineralization and antioxidant defense processes were revealed upon sponge-induced shell damage [21]. Nevertheless, infestation by the boring sponge constitutes a chronic stressor, wherein shell damage occurs concurrently with nutrient competition with the sponge and shifts in the oyster microbiome [14]. In this regard, it remains unclear to what extent the observed changes in immune parameters are directly related to shell damage and subsequent repair. Investigating the response to a single damage allows the contribution of this factor to be assessed separately from the factors associated with chronic exposure to the boring sponge.

Thus, the aim of the present study was to evaluate the effect of mechanical shell damage on the temporal dynamics of cellular and molecular parameters of the innate immune response in the Pacific oyster *M. gigas* during shell repair. For this purpose, we measured reactive oxygen and nitrogen species production, phagocytic activity, hemocyte adhesion and aggregation, and the expression of immunity-associated genes in the mantle during the 14 days post-damage.

## 2. Materials and Methods

### 2.1. Animal and Experimental Design

A total of 160 Pacific oysters (5 years old; body mass 97.7 ± 5.1 g; shell length 18.2 ± 2.4 cm) were collected in March of 2025 from the LLC “Mariculture” mariculture farm. At the time of collection, seawater temperature was 13–15 °C, salinity 18‰, pH 8.0, and dissolved oxygen concentration in the range of 7–8 mg L^−1^. Oysters were transported to the laboratory in insulated containers without water to maintain humidity and minimize transportation-related stress. Upon arrival, animals were placed in eight 50 L plastic tanks at a density of 20 individuals per tank. The tanks were filled with filtered artificial seawater, and the oysters were maintained under the following conditions: salinity 18‰, pH 7.8, temperature 15 ± 0.5 °C, continuous aeration, and a 12 h light:12 h dark photoperiod. These conditions were selected to approximate those recorded at the collection site. The oysters were acclimated to laboratory conditions for two weeks before the start of the experiment. During acclimation, animals were fed twice daily with a mixed algal suspension of *Tetraselmis viridis* and *Chlorella vulgaris* f. *suboblonga* (1:1 biomass ratio) to maintain a final cell density of approximately 4 × 10^5^ cells mL^−1^. Thirty percent of the water in each tank was renewed every 48 h, while uneaten food and biodeposits were removed daily.

### 2.2. Sample Collection

Following acclimation, oysters were randomly assigned to two experimental groups (*n* = 80 per group): a control group comprising intact individuals and an experimental group subjected to standardized mechanical shell damage. Each experimental group consisted of four replicate 50 L tanks containing 20 oysters per tank. The experimental shell damage was produced by creating multiple small circular defects in both shell valves. Thirty defects, approximately 1–2 mm in diameter, were created on each valve using a small electric dental handpiece equipped with a round dental bur with a working-head diameter of 1.6 mm. The defects were distributed within a shell region extending from the hinge toward the adjacent shell area and occupying approximately one-third of the surface area of each valve. This estimate refers to the shell region over which the defects were distributed rather than to the cumulative surface area of the individual defects. Drilling was limited to the superficial shell layers and stopped before the inner shell surface was reached. The integrity of the inner shell surface was confirmed by visual inspection after drilling. The entire shell-damage procedure lasted no more than 5 min. During the procedure, control oysters were kept adjacent to the shell-damaged oysters and were subjected to the same handling, duration of air exposure, ambient drilling noise, and environmental vibration, but their shells were not drilled. After the procedure, oysters from both groups were returned to aerated tanks. Mortality was monitored throughout the 14-day experimental period. Mortality remained below 2% in both the control and shell-damaged groups. Deceased oysters were promptly removed from the tanks and excluded from subsequent sampling.

Hemolymph and mantle tissue were sampled from control and experimental groups according to different sampling schedules. Hemolymph was collected on days 1, 2, 7, and 14 post-damage for the assessment of hemocyte functional parameters, whereas mantle tissue was collected on days 1, 7, and 14 post-damage for gene expression analysis. These tissues were selected because hemocytes are major cellular effectors of the innate immune response, whereas the mantle is directly involved in shell formation and repair [27,28]. For hemolymph collection, approximately 1 mL of hemolymph was withdrawn from the pericardial cavity using a sterile syringe equipped with a 25-gauge needle, immediately placed on ice, and processed within 30 min for subsequent analysis of hemocyte functional parameters. Mantle tissue samples were excised from the area adjacent to the damage site in damaged oysters and from the corresponding mantle region in control animals. The samples were immediately frozen and stored at −80 °C until RNA isolation. Sampling from control and experimental groups was performed in parallel at each time point to minimize potential effects of sampling time and laboratory maintenance on the measured parameters. At each sampling time point, independent subgroups of animals were sampled; no repeated measurements were performed on the same individuals. All procedures involving animals were conducted in accordance with institutional guidelines for the care and use of aquatic organisms.

### 2.3. Assessment of Intracellular ROS and NO Production

Intracellular production of reactive oxygen species (ROS) and reactive nitrogen species (RNS) in hemocytes was quantified by flow cytometry using the fluorescent probes 2′,7′-dichlorodihydrofluorescein diacetate (DCF-DA; Sigma-Aldrich, St. Louis, MO, USA) and 4-amino-5-methylamino-2′,7′-difluorofluorescein diacetate (DAF-FM DA; Lumiprobe RUS Ltd., Moscow, Russia), respectively. DCF-DA was used as an indicator of intracellular ROS production. Following cellular uptake, endogenous esterases hydrolyze DCF-DA to 2′,7′-dichlorodihydrofluorescein (DCFH), which is subsequently oxidized to the fluorescent product 2′,7′-dichlorofluorescein (DCF) (excitation/emission maxima: 485/530 nm). Intracellular RNS production was assessed using DAF-FM DA. Following cellular uptake and intracellular deacetylation, DAF-FM forms a fluorescent triazole product in the presence of NO-derived reactive nitrogen species (excitation/emission maxima: 495/515 nm).

For each assay, 1 mL of hemocyte suspension (adjusted to a density of approximately 1 × 10^6^ cells mL^−1^) was incubated with DCF-DA or DAF-FM DA at a final concentration of 10 µM for 40 min at room temperature in the dark. Following incubation, cells were kept on ice and analyzed immediately by flow cytometry. Fluorescence was measured using a MACSQuant Analyzer 10 flow cytometer (Miltenyi Biotec GmbH, Bergisch Gladbach, Germany) equipped with a 488 nm air-cooled solid-state laser. For each sample, 10,000 events were recorded, and intracellular ROS and RNS levels were expressed as mean fluorescence intensity (MFI). Measurements were performed in unstimulated hemocyte suspensions and therefore represent spontaneous intracellular ROS and RNS production.

### 2.4. Phagocytosis

The phagocytic activity of hemocytes was assessed using Neutral Red-stained zymosan particles according to the method described by Ivanina et al. [29], with modifications. Briefly, heat-stabilized zymosan particles (Sigma-Aldrich, St. Louis, MO, USA) were pre-stained with Neutral Red and added to hemocyte suspensions at a ratio of 200 zymosan particles per hemocyte. Following a 30-min incubation at room temperature with gentle agitation, samples were centrifuged (1000× *g*, 10 min) and washed twice with artificial seawater (ASW; salinity 18‰) to remove non-phagocytosed zymosan particles. Zymosan suspensions of known particle concentrations processed under the same conditions were used to generate a calibration curve. To quantify phagocytosed zymosan, hemocyte pellets and calibration standards were lysed in 1% acetic acid in 50% ethanol for 5 min, and Neutral Red absorbance was measured at 550 nm using a microplate spectrophotometer (SuPerMax 3100, Shanghai Flash Spectrum Biotechnology Co., Ltd., Shanghai, China). The amount of phagocytosed zymosan was determined from the calibration curve and expressed as the number of zymosan particles internalized per 100 hemocytes.

### 2.5. Hemocyte Adhesion and Aggregation Assay

Hemocyte adhesion and aggregation were assessed using a method adapted from Aladaileh et al. [30] and Tkachuk et al. [31]. Briefly, 30 µL of hemocyte suspension was placed on a glass microscope slide and incubated for 10 min at room temperature to allow hemocyte attachment and aggregation. The samples were then fixed and stained with eosin and methylene blue according to the May–Grünwald staining procedure (Abris, Moscow, Russia) for 2 min. Stained preparations were examined using an Olympus CX43 microscope (Olympus Corporation, Tokyo, Japan) in transmitted-light mode. Ten randomly selected visual fields were imaged for each sample and analyzed using ImageJ software v1.5. The numbers of adherent hemocytes and hemocyte aggregates were quantified in each visual field. The results were expressed as the mean number of adherent hemocytes and aggregates per visual field.

### 2.6. RNA Expression Analysis

Gene expression analysis included the immune-related genes *IL-17-3*, *IL-17-6*, *TNF-1*, *TNF-2*, and *Toll-1*. These genes were selected based on their previously reported involvement in the immune response of *M. gigas*. *Toll-1* is a Toll-like receptor whose expression responds to bacterial challenge [32]. *IL-17-3* and *IL-17-6* were selected as members of the expanded *IL-17* family that differ in their transcriptional responses to bacterial immune stimuli: *IL-17-3* is strongly induced by LPS and *Vibrio* challenge, whereas *IL-17-6* responds to peptidoglycan [33]. *TNF-1* and *TNF-2* were selected based on their reported effects on oyster immune functions. *TNF-1* regulates hemocyte responses, including phagocytosis, whereas *TNF-2* is involved in antibacterial activity and nitric production [34,35]. In addition to their roles in pathogen defense, these immune-related genes are implicated in damage-induced inflammatory signaling, tissue repair, and the modulation of hemocyte dynamics. Given that shell damage initiates energetically costly repair processes involving hemocyte migration to the mantle, assessing the expression of these specific cytokine and receptor genes helps determine whether mechanical damage independently triggers a mucosal inflammatory cascade in the underlying mantle tissue. Total RNA was isolated from mantle tissue of *M. gigas* using the RNeasy Mini Kit (Qiagen, Hilden, Germany) according to the manufacturer’s protocol. Ten mantle samples from each experimental group were analyzed at each of the three sampling times (days 1, 7, and 14 post-damage; *n* = 10 per group per time point). Extracted RNA was treated with DNase I for 15 min at 37 °C and resuspended in 50 µL of nuclease-free water. RNA integrity was assessed by electrophoresis in a 1.5% agarose gel stained with ethidium bromide, and RNA concentration was measured using a Qubit 4.0 fluorometer (Thermo Fisher Scientific, Waltham, MA, USA). cDNA was synthesized from 500 ng of total RNA using the MMLV reverse transcription kit (Evrogen, Moscow, Russia) according to the manufacturer’s protocol, in a final reaction volume of 15 µL. Gene expression analysis included the immune-related target genes *IL-17-3*, *IL-17-6*, *TNF-1*, *TNF-2*, and *Toll-1* receptor. Primer sequences used for RT-qPCR analysis are listed in Table 1.

Quantitative real-time PCR (RT-qPCR) was performed using a CFX 96 Real-Time PCR System (Bio-Rad Laboratories, Hercules, CA, USA) and BioMaster SYBR Blue qPCR mix (Biolabmix, Novosibirsk, Russia). Each reaction was performed in a final volume of 25 µL containing 0.5 µL of cDNA, 0.5 µM of each primer, qPCR mix, and nuclease-free water. Thermal cycling conditions consisted of an initial denaturation at 95 °C for 30 s, followed by 37 cycles of denaturation at 95 °C for 10 s, annealing at 60 °C for 10 s, and extension at 72 °C for 15 s. Melting-curve analysis was performed from 55 to 95 °C to verify amplification specificity. All samples were analyzed in technical triplicate, and no-template controls were included in each RT-qPCR run. Quantification cycle (Cq) values were determined using CFX 96 software (v. 3.1). Amplification efficiencies were determined from standard curves generated using serial dilutions of cDNA. The expression stability of *EF-1 α* was evaluated using BestKeeper v.1 [38], and *EF-1 α* was subsequently used for normalization of the expression levels of the target genes. Relative gene expression was calculated using the 2^−ΔΔCt^ method [39], with *EF-1 α* as the reference gene. Expression values are presented as fold changes relative to the control group on day 1.

### 2.7. Statistical Analyses

Statistical analyses were performed using RStudio version 4.6.1. Hemocyte functional parameters (intracellular ROS and RNS production, phagocytic activity, adhesion, and aggregation) were analyzed using two-way analysis of variance (ANOVA), with treatment (control vs. shell damage) and time (1, 2, 7, and 14 days post-damage) as fixed factors, including the treatment × time interaction. To account for potential temporal variation in hemocyte functional parameters unrelated to shell damage, control and shell-damaged oysters were sampled in parallel at each time point. Accordingly, pairwise comparisons were performed between the control and shell-damaged groups within each sampling day rather than against a single baseline control. This approach allowed us to evaluate shell damage-associated differences relative to the corresponding time-matched controls, thereby minimizing the potential confounding influence of background temporal variability. *p*-values from pairwise comparisons were adjusted for multiple comparisons using the Holm method. Hemocyte data are presented as means with parametric 95% confidence intervals (95% CIs). This approach was chosen because 95% CIs provide an estimate of the precision and uncertainty associated with the sample mean, whereas standard deviation (SD) describes the variability of individual observations. The 95% CIs were calculated using the sample mean, the standard error of the mean (SEM), and the critical value of Student’s t-distribution with n−1 degrees of freedom, where n represents the number of biological replicates.

Gene expression data (*TNF-1*, *TNF-2*, *Toll-1*, *IL-17-3*, and *IL-17-6*) were log-transformed prior to statistical analysis and analyzed using a bootstrap approach with 10,000 resamples. Pairwise comparisons between control and shell-damaged groups at each sampling time were performed using bootstrap-derived *p*-values, with adjustment for multiple comparisons using the Holm method. Gene expression data are presented as means with non-parametric bootstrap 95% confidence intervals based on 10,000 resamples. The statistical unit for all analyses was the individual animal (*n* = 10 independent oysters per group per time point). Differences were considered statistically significant at *p* < 0.05.

## 3. Results

### 3.1. Intracellular ROS and RNS Production

On days 1 and 2 after mechanical shell damage, the level of intracellular ROS in *M. gigas* hemocytes was significantly lower compared to the control group (*p* < 0.05; Figure 1a). Starting from the 7th day, no significant differences in ROS levels were found between the control and the shell-damaged group (*p* > 0.05). Throughout the experimental period, no significant intergroup differences were recorded for RNS levels (*p* > 0.05; Figure 1b).

### 3.2. Phagocytic Activity

At 1 day post-damage, phagocytic activity of *M. gigas* hemocytes was significantly elevated relative to the control (*p* < 0.05; Figure 2). By day 2, an opposite trend was evident, with phagocytic activity in the damaged group being significantly reduced compared to the control (*p* < 0.05). From day 7 onwards, no statistically significant differences between the control and damaged groups were observed (*p* > 0.05).

### 3.3. Adherent Cells and Aggregation

On days 1, 2, and 7 after mechanical shell damage, hemocyte adhesion in *M. gigas* was significantly lower compared to the control group (*p* < 0.05; Figure 3a). By day 14, no statistically significant differences between the groups were detected (*p* > 0.05). Hemocyte aggregation in the damaged group was significantly lower than in the control on days 1 and 7 (*p* < 0.05; Figure 3b). In contrast, on day 14, a reversed trend was evident, with hemocyte aggregation in shell-damaged oysters being significantly higher than in the control (*p* < 0.05).

### 3.4. Gene Expression of Immune-Related Markers

On day 7 after mechanical shell damage, the expression level of the *TNF-2* gene in the mantle tissues of *M. gigas* was significantly lower compared to the control group (*p* < 0.05; Figure 4). For the *TNF-1*, *Toll-1*, *IL-17-3*, and *IL-17-6* genes, no statistically significant differences between the control and shell-damaged groups were detected at any of the time points examined (*p* > 0.05).

## 4. Discussion

Shell repair represents a complex physiological process involving mantle cells and hemocytes, which are engaged in both biomineralization and immune defence [24]. The results obtained in the present study demonstrate that mechanical shell damage in *M. gigas* is accompanied by alterations in a number of cellular and molecular parameters of innate immunity. Most of the observed changes were restricted to the early stages of shell repair.

One of the earliest responses to shell damage was a decrease in ROS production by hemocytes: on days 1 and 2, ROS levels in the experimental group were lower than in the control, whereas from day 7 onwards, no differences between the groups were observed. ROS are an important component of the cellular immune response in bivalves and are involved in the killing of phagocytosed microorganisms [40,41,42]. At the same time, their production is closely linked to the metabolic state of cells, since one of the main sources of ROS is the mitochondrial electron transport chain [43]. Therefore, the decrease in ROS production during the first two days after damage may be related not only to changes in the effector activity of hemocytes, but also to alterations in their metabolic state during the early repair period. A decline in ROS production has previously been described in bivalves under exposure to various stressors. A decrease in ROS generation has been reported by Adzigbli et al. [44] in cells of the Pacific oyster under acute hypoxia and during reoxygenation, which the authors attributed to LEAK respiration. A decrease in ROS production has also been observed in bivalves exposed to hypoxia and acidification, which has been attributed to general suppression of metabolic and immune functions [45,46]. Shell repair is an energy-demanding process, and the observed decrease in ROS production may also be associated with changes in energy allocation during early repair. Since both ROS generation and shell matrix synthesis require metabolic resources, reduced ROS production may reflect a shift in resource allocation towards shell restoration. However, this explanation remains hypothetical, as energy metabolism was not directly assessed in the present study. By contrast, in *M. gigas* infested with boring sponges, the opposite trend was observed, with hemocytes exhibiting elevated ROS production [21,25]. The differences between these findings may be related to the duration and nature of the damaging exposure. In the present experiment, mechanical damage was inflicted once, after which shell repair occurred. In the case of boring sponge infestation, shell damage is chronic and continues throughout the period of host-sponge interaction. Moreover, infestation by *Pione vastifica* is accompanied by changes in the gill microbiome and damage to oyster tissues [14]. Under these conditions, prolonged damage combined with changes in the microbial environment may sustain hemocyte activation and increased ROS production. ROS and RNS levels also varied over time in the control group. Such variation may be related to natural physiological changes or responses to laboratory conditions [47,48,49]. The underlying causes were not investigated in the present study. However, parallel sampling of control and shell-damaged oysters allowed us to evaluate the effects of shell damage relative to the corresponding control at each time point, reducing the potential influence of background temporal variation.

In contrast to ROS, RNS production did not differ from the control at any of the time points examined following mechanical shell damage. The differential response of these parameters may be related to the specific mechanisms of their generation in hemocytes. While ROS production is largely linked to mitochondrial metabolism and cellular respiration activity, the formation of RNS is primarily determined by NO-dependent reactions involving nitric oxide synthase [40].

In addition to changes in ROS production, shell damage affected the capacity of hemocytes for phagocytosis, adhesion, and aggregation. At 1 day post-damage, phagocytic activity was elevated above control levels, whereas both adhesion and aggregation were reduced. On day 2, phagocytic activity decreased relative to the control, concomitant with a decline in adhesion. Changes in adhesion persisted up to day 7; at the same time point, hemocyte aggregation was also reduced. By day 14, no differences between groups were detected for phagocytic activity and adhesion, whereas hemocyte aggregation in the damaged group exceeded control values. Phagocytosis, adhesion, and aggregation are functionally related cellular responses that depend on cytoskeletal reorganization [50,51]. Elevated cytosolic Ca^2+^ is involved in actin cytoskeleton reorganization, which is required for hemocyte adhesion, migration, and phagocytosis [52,53,54]. Reparation requires Ca^2+^ delivery to the site of damage and its incorporation into the forming mineral layer, and hemocytes are considered as one of the cellular components involved in the transport and deposition of mineral material [16,55,56]. Since Ca^2+^ is involved in both biomineralization and cytoskeleton-dependent hemocyte functions, changes in calcium distribution during shell repair may contribute to the observed alterations in hemocyte activity. However, calcium-related parameters were not measured in the present study, and this possibility requires further investigation.

In addition to changes in hemocyte functional activity, we evaluated the expression of immune genes in the mantle tissue adjacent to the damaged area. The mantle is directly involved in shell formation and reparation and, at the same time, represents one of the tissues that are in contact with the external environment and participate in the immune defence of molluscs [17]. In the present study, the expression of most of the immune genes examined in the mantle tissue—*IL-17-3*, *IL-17-6*, *TNF-1*, and *Toll-1*—did not differ from the control following shell damage. The absence of changes in these genes does not preclude a possible earlier response of the corresponding genes to damage. In experimental shell damage of *Pinctada fucata martensii*, changes in immune signalling pathways in the outer mantle were observed mainly in the first hours after exposure [17]. Specifically, changes in several immune signalling pathways were observed as early as 4–12 h after damage. A rapid response to shell damage was also shown in *M. gigas*: the expression of tissue inhibitors of metalloproteinases (*TIMPs*) in circulating hemocytes began to increase at 3–6 h, reached a peak at approximately 9 h, and then gradually returned to baseline levels [57]. In the present work, the first expression analysis was performed at 24 h, so short-term changes occurring prior to this time point were not assessed.

In contrast to the other genes examined, the expression of *TNF-2* decreased on day 7 after shell damage and returned to control levels by day 14. *TNF-2*, like *TNF-1*, belongs to the family of *TNF*-like cytokines in oysters [35]. However, the amino acid sequence identity between *TNF-1* and *TNF-2* is only 33% [35], which has previously been considered a possible indication of functional diversification within the *TNF* family [34,35]. Differences among family members were also observed in our experiment: *TNF-1* expression did not change, whereas *TNF-2* decreased on day 7. Changes in *TNF* expression after mechanical damage have been previously shown in *Haliotis discus discus*, in which damage to the shell and mantle edge was accompanied by an increase in *TNF-α* expression in the mantle during the first hours after exposure [58]. *TNF-2* has been shown to be involved not only in the regulation of immune effector responses, but also in hemocyte proliferation via Runx and cell cycle genes [35,59]. However, the functional significance of the decreased *TNF-2* expression in the mantle tissue on day 7 after shell damage remains unclear. However, the present study focused on later stages of the response to shell damage, whereas earlier sampling times (e.g., 2, 6, and 12 h) would be necessary to characterize the initial transcriptional response.

## 5. Conclusions

Our findings demonstrate that shell integrity disruption in *Magallana gigas* is not limited to local damage of the protective structure, but is accompanied by changes in the physiological state of the oysters. The bidirectional dynamics of cellular parameters and the absence of changes in the expression of four out of five immune genes examined do not indicate a general suppression of immune function following shell damage. Rather, the response was characterised by transient alterations in individual cellular reactions, whereas at the level of the mantle genes studied, changes were restricted to a decrease in *TNF-2* expression on day 7. Thus, the state of the shell should be considered as one of the factors affecting the functional status of cultured oysters. Even a single non-penetrating damage induces a temporary reorganization of cellular defence responses, and some changes in cellular parameters and *TNF-2* expression persisted at later stages of repair. These features should be taken into account when assessing the condition of oysters after mechanical damage occurring under cultivation conditions.

## Figures and Tables

**Figure 1:**
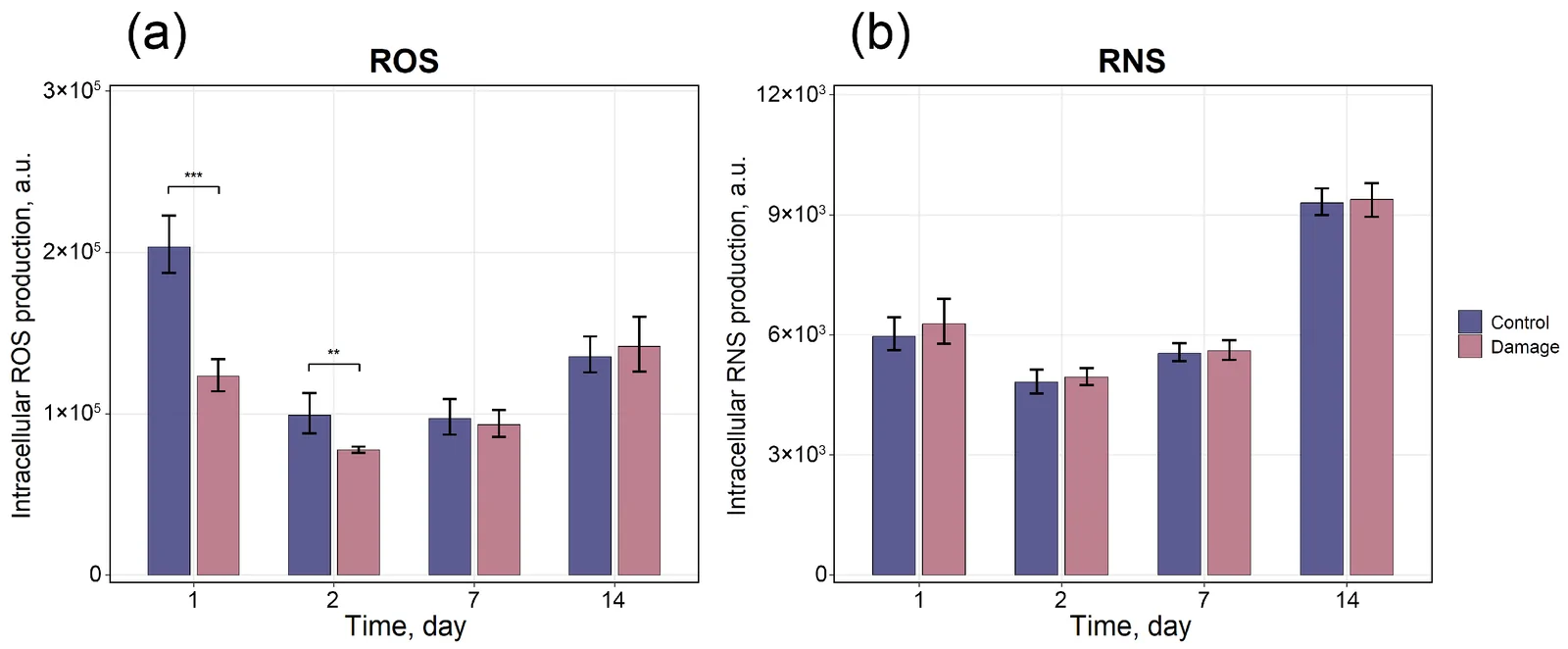
Intracellular production of reactive oxygen species (ROS) (**a**) and reactive nitrogen species (RNS) (**b**) in hemocytes of *Magallana gigas* in the control and shell-damage groups at 1, 2, 7, and 14 days post-damage. Data are presented as means with 95% confidence intervals. Asterisks indicate statistically significant differences between the control and shell-damage groups at the corresponding sampling time (** *p* < 0.01; *** *p* < 0.001; two-way ANOVA followed by pairwise comparisons with Holm adjustment; *n* = 10).

**Figure 2:**
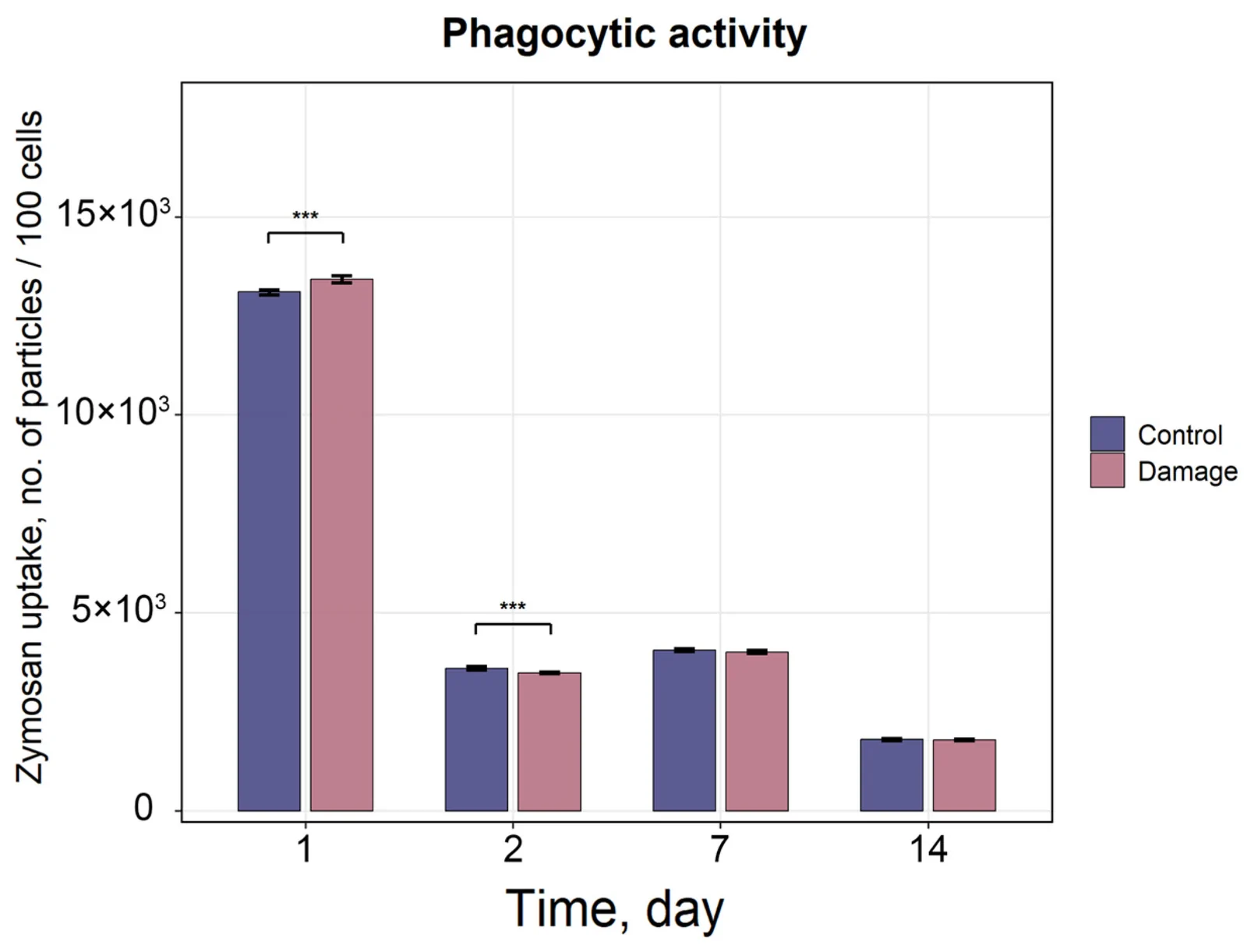
Phagocytic activity of hemocytes of *Magallana gigas* in the control and shell-damage groups at 1, 2, 7, and 14 days post-damage. Phagocytic activity is expressed as the number of zymosan particles internalized per 100 hemocytes. Data are presented as means with 95% confidence intervals. Asterisks indicate statistically significant differences between the control and shell-damage groups at the corresponding sampling time (*** *p* < 0.001; two-way ANOVA followed by pairwise comparisons with Holm adjustment; *n* = 10).

**Figure 3:**
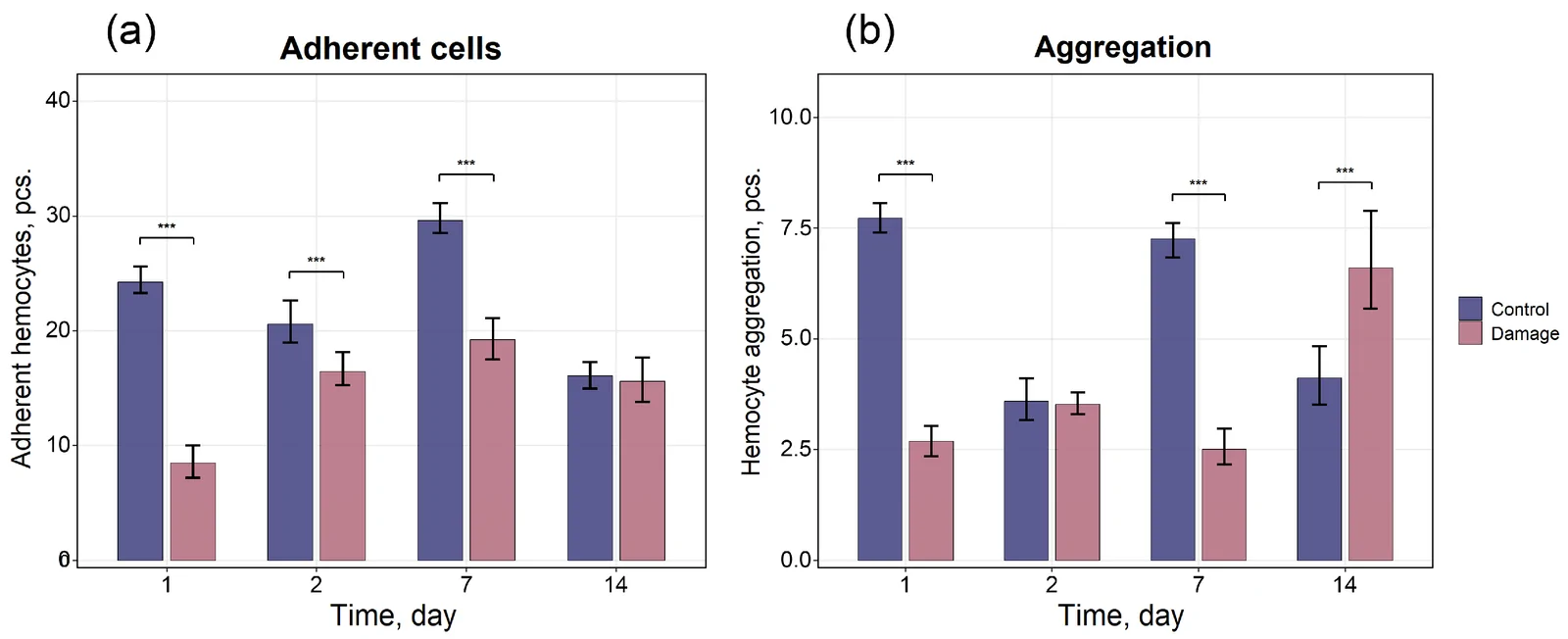
Adhesion (**a**) and aggregation (**b**) of hemocytes of *Magallana gigas* in the control and shell-damage groups at 1, 2, 7, and 14 days post-damage. Adhesion and aggregation are expressed as the mean numbers of adherent hemocytes and hemocyte aggregates per visual field, respectively. Data are presented as means with 95% confidence intervals. Asterisks indicate statistically significant differences between the control and shell-damage groups at the corresponding sampling time (*** *p* < 0.001; two-way ANOVA followed by pairwise comparisons with Holm adjustment; *n* = 10).

**Figure 4:**
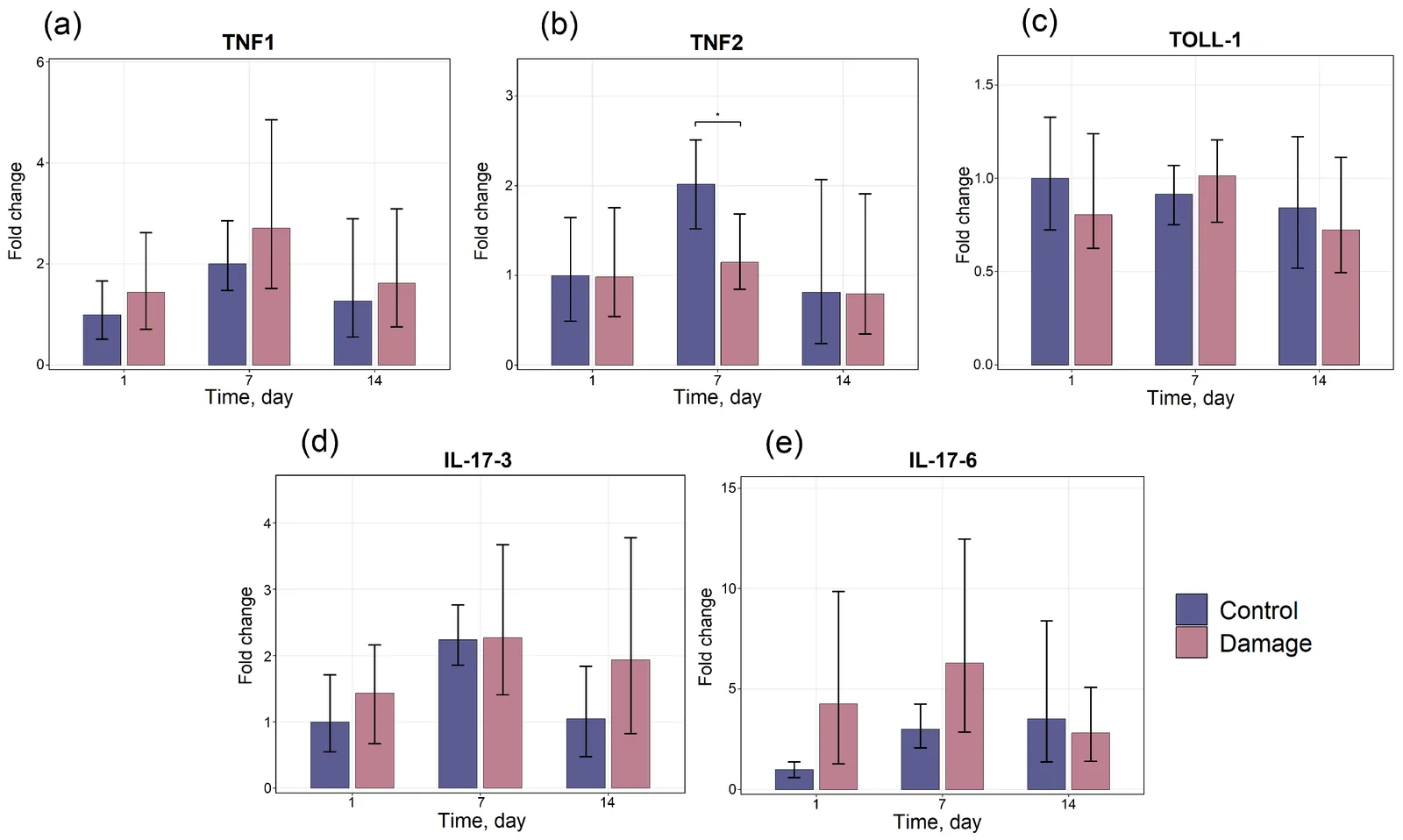
Tumor necrosis factors *TNF-1* (**a**), *TNF-2* (**b**); Toll-like receptor *Toll-1* (**c**) and *IL-17-3* (**d**), *IL-17-6* (**e**) in the control and shell-damage groups at 1, 7, and 14 days post-damage. Gene expression was normalized to *EF-1α* and expressed as fold change relative to the control group on day 1. Data are presented as means with bootstrap 95% confidence intervals based on 10,000 resamples. Asterisks indicate statistically significant differences between the control and shell-damage groups at the corresponding sampling time (* *p* < 0.05; bootstrap pairwise comparisons with Holm adjustment; *n* = 10).

**Table 1:** Primer sequence of target and reference genes for RT-qPCR.

Gene	Sequence (5′-3′)	Reference	Title
*IL-17-3*	F: TCACTACCAACTGAACTACGACCG R: TGCGACAGAGCCTGAGAACC	[36]	interleukin-17, a proinflammatory cytokine
*IL-17-6*	F: CCATTTGTCCGACCTACCGT R: CCACCCTGCGTAGAACCATC	[36]	interleukin-17, a proinflammatory cytokine
*TNF-1*	F: CTTCTCGTCTGCGGCTTCTTT R: CAGGGCTGCGGTCTTTCC	[36]	tumor necrosis factor, a proinflammatory cytokine
*TNF-2*	F: CCACTACGCCCACTAAGAAACTG R: AAGCGACCATTGCGAAACTCCAT	[36]	tumor necrosis factor, a proinflammatory cytokine
*Toll-1*	F:AAGTTCTCGTGAAGTCCCATCC R:ATACAGGCGTCTCGTAACCATC	[32]	Toll-like receptor
*EF-1 α*	F:AGTCACCAAGGCTGCACAGAAAG R:TCCGACGTATTTCTTTGCGATGT	[37]	elongation factor

## Data Availability

The datasets generated and/or analyzed during the current study are available from the corresponding author on request.

## Abbreviations

The following abbreviations are used in this manuscript:

ANOVA	Analysis of variance
ASW	Artificial seawater
Cl	Confidence interval
Cq	Quantification cycle
DAF-FM DA	4-amino-5-methylamino-2′,7′-difluorofluorescein diacetate
DCF	2′,7′-dichlorofluorescein
DCF-DA	2′,7′-dichlorodihydrofluorescein diacetate
DCFH	2′,7′-dichlorodihydrofluorescein
MFI	Mean fluorescence intensity
RNS	Reactive nitrogen species
ROS	Reactive oxygen species
